# The relationship between family-school socioeconomic status match and adolescent aggressive behavior

**DOI:** 10.3389/fpsyg.2024.1407851

**Published:** 2024-07-05

**Authors:** Furong Lu, Yuyu Wang, Xinru Wu

**Affiliations:** ^1^School of Education Science, Shanxi University, Taiyuan, China; ^2^Centre for Psychological Health Education, Henan University of Science and Technology, Luoyang, China

**Keywords:** family SES, school SES, adolescent, aggressive behaviors, parent–child relationship

## Abstract

The objective of the present study was to analyze the effect of the match between family and school socioeconomic status (SES) on adolescents’ aggressive behaviors. Additionally, the moderating roles of gender and the parent–child relationship were examined. A total of 2,823 adolescents completed the Aggressive Behavior Scale, the Parent–Child Relationship Scale, and the Family SES Scale. School SES was measured by the average family SES of all students in the school. SES was categorized as high or low based on one standard deviation above or below the mean. The results showed that when there was a match between family and school SES, adolescents with “Low Family-Low School” SES exhibited more aggressive behaviors compared to those with “High Family-High School” SES. When there was a mismatch between family and school SES, adolescents with “High Family-Low School” SES exhibited higher levels of aggressive behaviors than those with “Low Family-High School” SES. Gender did not moderate these effects. Furthermore, when the parent–child relationship was poor, adolescents exhibited higher levels of aggressive behaviors when family SES exceeded school SES. Conversely, the effects of family and school SES on aggressive behavior were not significant when the parent–child relationship was strong. The present study highlights that the match and mismatch between family and school SES significantly influence adolescents’ aggressive behaviors and that a strong parent–child relationship has a protective effect.

## 1 Introduction

Aggressive behavior can have a significant impact on adolescents and has been recognized as a major public safety problem worldwide (Li et al., 2012). This issue has attracted considerable interest in psychology research (Chen et al., 2018; Geng et al., 2018; Lin et al., 2021) and is defined as the act of directly or indirectly causing bodily or psychological harm to another person (Ji and Zhang, 2007). Aggressive behavior not only causes harm to others but also has negative effects on the aggressors themselves (Gini et al., 2014; Jia et al., 2016). Therefore, identifying risk factors for aggressive behavior is crucial.

Based on ecosystem theory (Bronfenbrenner and Morris, 1998), the socioeconomic status (SES) of the family and school are important for adolescents, as these can be considered microsystems. Socioeconomic status is defined by Bradley and Corwyn (2002) as the specific position individuals or groups occupy in society based on the social resources they possess. Matthews and Gallo (2011) provided a clear explanation of these social resources, stating that they include valuable resources such as education, wealth, and social status enjoyed by families. Family socioeconomic status, reflecting these resources, ranks families hierarchically and indicates the differences in resources that each family can currently access or may access in the future. This status specifically refers to the rank, level, and status of a family in society. It has been found that adolescents who grow up in environments with low family SES exhibit more aggressive tendencies (Greitemeyer and Sagioglou, 2016). School SES refers to the overall level of socioeconomic status of the student population in the school. Research indicates that higher school SES is associated with better academic performance among students (Xie and Zhang, 2018; Xue et al., 2020). Additionally, peer aggression tends to decrease when the SES of the school is high (López et al., 2021).

As the two primary environments in adolescents’ lives, the relationship between family-school socioeconomic status and adolescents’ aggressive behaviors deserves to be explored. Only two studies have examined the effect of the interaction between family and school SES on outcomes using multilevel analysis (Zhang et al., 2016; Xue et al., 2020). Xue et al. (2020) demonstrated that the SES of both the family and the school is positively correlated with students’ individual scores in mathematics and Chinese language. Furthermore, school SES strengthened the relationship between family SES and mathematics scores among students in rural schools. In contrast, the interaction effect between family and school SES was not significant in urban areas. Similarly, Zhang et al. (2016) found that children’s academic success in mathematics is positively predicted by both family and school SES. Children’s academic ability in mathematics was strongly and positively predicted by school SES even after family SES was taken into account, with a high impact size. Prior studies have shown that both family and school SES, as well as their interaction, can influence students’ academic performance. However, more research is needed to understand the impact of family and school SES on individual behavior, particularly the potential effects of their matching on students’ behavior and psychology. Therefore, this study uses response surface analysis (Edwards, 2002) to provide a more thorough and in-depth investigation of the socioeconomic status of the family and the school. The purpose of the present study is to analyze the relationship between the match of family and school SES and adolescents’ aggressive behaviors. Additionally, we explored the moderating roles of the parent–child relationship and gender.

### 1.1 The link between discrepancies in family-school SES and adolescent aggressive behaviors

Although family and school SES are crucial factors for adolescents, previous studies have overlooked the impact of matching family-school SES on adolescents’ aggression. In recent years, numerous news articles in China have highlighted the phenomenon of “school district housing,” where parents strive to enroll their children in prestigious schools. When choosing a school, parents may take into account the alignment of family and school SES. Disparities between adolescents’ family SES and school SES have been identified in previous research (Zhang et al., 2016). Based on these differences, four classifications emerge: “High Family-High School” (HF-HS) SES, “Low Family-Low School” (LF-LS) SES, “High Family-Low School” (HF-LS) SES, and “Low Family-High School” (LF-HS) SES. The former two classifications indicate matching SES types, while the latter two represent mismatching SES types.

When family and school SES are matched, a superimposed effect may occur. Family socioeconomic status is strongly associated with the developmental resources available to adolescents, with low socioeconomic status identified as a risk factor. School socioeconomic status is typically assessed through the average family socioeconomic status of all students in the school (Zhang et al., 2016). According to the cumulative risk model, the cumulative effect of “Low Family-Low School” (LF-LS) SES may influence adolescents’ aggressive behavior (Rutter, 1983). Cumulative risk has been shown to positively predict problematic and criminal behaviors (Doan et al., 2012; Lei et al., 2019). Research indicates that “LF-LS” SES has adverse effects on children’s development. For example, Greitemeyer and Sagioglou (2016) observed that individuals with lower socioeconomic status might perceive themselves as disadvantaged, potentially leading to aggressive responses. Xue et al. (2020) suggested, based on relative deprivation theory, that lower socioeconomic status both in the family and the school context correlates with poorer academic performance among students. Therefore, the current study hypothesizes that when there is a match between family and school SES, adolescents with “LF-LS” SES will exhibit more aggressive behavior compared to those with “HF-HS” SES. Furthermore, the challenge model of psychological resilience (Fergus and Zimmerman, 2005) suggests that for adolescents, prolonged exposure to either excessively low or excessively high risk factors can lead to significant negative developmental outcomes. Moderate exposure to risk factors over time, however, provides adolescents with an opportunity to learn how to adapt to their environment and manage crises. Thus, students with moderate levels of family and school SES may experience the lowest levels of aggression. Since no previous study has explored this concept of matchability, an exploratory analysis is conducted in this study.

When family and school SES are mismatched, adolescents with “LF-HS” SES tend to engage in upward comparisons. Such comparisons can lead to frustration, and individuals experiencing frustration may resort to direct or indirect aggression as a means of alleviation (Berkowitz, 1989). The low-status compensation theory (Henry, 2009) posits that the gap in status threatens the social value perception of lower SES groups, making them more sensitive and defensive when their values are threatened. Conversely, adolescents with “HF-LS” SES tend to engage in downward comparisons. According to social hierarchy theory (Fournier et al., 2002), individuals in higher positions within a hierarchical organization often exhibit aggressive behaviors to safeguard their superior status and secure preferential access to resources (Huhman, 2006). One study found that individuals from higher family SES demonstrate more aggressive behaviors (Dou et al., 2015). Therefore, when there is a mismatch between family SES and school SES, adolescents may exhibit aggressive behaviors regardless of whether they make upward or downward comparisons. Based on the above analysis, it is hypothesized that adolescents’ aggressive behavior increases when there is a mismatch between family and school socioeconomic status.

### 1.2 The moderating roles of gender and parent–child relationship

Gender differences have been a significant concern for researchers studying adolescent aggression. Studies have found that males tend to exhibit more physical aggressive behaviors than females, while females tend to display more relational aggressive behaviors (Ostrov and Godleski, 2010; Chen et al., 2019). However, some research has found no gender differences in aggressive behavior (Wang et al., 2019), and one study even reported lower levels of aggression in boys compared to girls (Ostrov and Godleski, 2010). These findings highlight inconsistency in the literature. Examining the disparities in aggressive behavior between boys and girls is crucial for developing more targeted prevention strategies. Therefore, the second goal of this study is to investigate whether there are gender differences in the effects of family-school SES matching on aggressive behavior.

Notably, the effect of the match between family and school socioeconomic status on aggressive behavior may be moderated by certain protective factors, such as the parent–child relationship. As the most fundamental relationship within the family, the parent–child relationship represents the first interpersonal connection individuals experience (Ling et al., 2018). The buffer theory (Aneshensel and Stone, 1982) suggests that a positive parent–child relationship, serving as a form of social support, acts as a protective mechanism for individuals (Yu et al., 2018). It can mitigate the negative effects of adverse stimuli, preventing individuals from experiencing various negative emotions and contributing to maintaining a healthy physical and mental state (Tian et al., 2018). Research has also found that a strong parent–child relationship can reduce the impact of parental corporal punishment on adolescents’ aggressive behaviors (Yu et al., 2018). In summary, a positive parent–child relationship may protect against the impact of a mismatch between family and school SES on aggressive behavior, whereas a negative parent–child relationship may exacerbate it. Therefore, exploring the moderating role of the parent–child relationship is the third aim of our study.

### 1.3 The present study

Our study aimed to investigate the correlation between family-school SES match and aggressive behavior. The moderating roles of gender and the parent–child relationship were also explored. To overcome the limitations of traditional methods, we employed polynomial regression and response surface analysis (Edwards, 2002). This approach provides a deeper understanding of the relationship between family SES, school SES, and aggressive behavior. The following hypotheses were proposed:

*H1:* When family-school SES matches, adolescents with “Low Family-Low School” SES exhibit more aggressive behaviors compared to those with “High Family-High School” SES.

*H2:* When family-school SES mismatches, adolescents may exhibit more aggressive behaviors compared to when family-school SES matches.

*H3:* Gender may moderate the impact of the match between family-school SES on aggressive behavior.

*H4:* The parent–child relationship may moderate the effect of family-school SES match on aggressive behavior.

## 2 Method

### 2.1 Participants

Convenient cluster sampling was used in this study to distribute 3,622 questionnaires and carry out surveys in Shanxi and Henan provinces, China. After excluding schools with insufficient participants and invalid questionnaires characterized by concentrated or patterned responses, 2,823 valid questionnaires were retained. The participants consisted of students from grades five to nine, with 46.1% male and 53.9% female. The number of students across these grades was balanced. The sample included students from thirteen elementary schools and twelve secondary schools, with 24.6% being only children. The average age of the participants was 13.38 years (*SD* = 1.56, range = 11-17 years). Among the students, 49.7% were aged 11–13 years, and 50.3% were aged 14–17 years. Moreover, 55.7% of the students lived in rural areas. The proportion of fathers with a college education (including junior college and above) was 17%, while for mothers, it was 16.2%.

### 2.2 Measures

#### 2.2.1 Family SES and school SES

Fathers’ and mothers’ education levels, along with their total family income, were used to indicate the family’s socioeconomic status (SES) in the study. Parents reported their education levels on a 9-point scale (1 = did not go to school, 9 = master’s degree and above). Family total income was reported by mothers in response to the question: “What is your monthly household income?” Income levels were reported on a 9-item scale (1 = less than RMB2,000, 9 = more than RMB16,001). Following the synthesis method of Ryabov and Van Hook (2007), family income and parents’ education level were standardized before being added together. This study adopted the method used by Zhang et al. (2016), where the school’s socioeconomic status was typically determined by the average family socioeconomic status of all students in the school. The final calculations in this study resulted in the following proportions: “High Family-High School” (HF-HS) is 51.65%, “Low Family-Low School” (LF-LS) is 14.10%, “High Family-Low School” (HF-LS) is 23.98%, and “Low Family-High School” (LF-HS) is 10.27%.

#### 2.2.2 Aggressive behavior

The Adolescent’s Aggressive Behavior Scale (Dong and Lin, 2011) was utilized in this study. The questionnaire consists of 10 items measuring two dimensions: physical aggression and indirect aggression. Each item was rated on a 4-point scale (1 = never, 4 = often). The Cronbach’s Alpha coefficient for this scale was 0.84. Based on a two-dimensional model, the validity of the scale was verified, and confirmatory factor analysis (CFA) indicated good data fit: *χ*^2^/*df* = 3.60, NFI = 0.99, IFI = 0.99, TLI = 0.98, CFI = 0.95, RMSEA = 0.03.

#### 2.2.3 Parent–child relationship

The questionnaire developed by the National Children’s Study of China (NCSC) was used in this study (Dong and Lin, 2011). The parent–child relationship was reported by the students. The questionnaire consists of 23 items and assesses both positive and negative aspects of the parent–child relationship. These items are divided into eight dimensions: satisfaction, worry, closeness, emotion, conflict, instrumental support, value affirmation and companionship. Each item was rated on a 5-point scale (1 = never, 5 = often). For the “Worry” and “Conflict” dimensions, reverse scoring was required. Subsequently, the total score of all items was calculated, with higher scores indicating a better parent–child relationship. The Cronbach’s Alpha coefficient for this scale was 0.88. The validity of the scale was confirmed based on an eight-dimensional model, and confirmatory factor analysis (CFA) indicated good data fit: *χ*^2^/*df* = 10.40, NFI = 0.98, RFI = 0.93, IFI = 0.99, TLI = 0.94, CFI = 0.99, RMSEA = 0.05.

### 2.3 Procedure

This study was approved by the ethics committee of the first author’s university. Participants were briefed on the study’s objectives and precautions before completing the questionnaire. Parental consent and student assent were obtained from all participants. With the school’s consent, the test was conducted by psychology graduate students who had received training. Responses from the students were collected immediately after completing the questionnaires.

### 2.4 Data analyses

Traditionally, the primary method for objectively measuring consistency is the difference score. However, it has five issues: reduced reliability, difficulties in variable explanation, complex coefficient interpretation, lack of parameter constraints, and reduced variable dimensions (Tao and Cao, 2020). Edwards (2002) proposed replacing the traditional difference-score approach with polynomial regression and response surface analysis, which can address these deficiencies. Based on the focus and objectives of our study, conducting polynomial regression analysis will enable us to accurately examine the relationship between family and school socioeconomic status and adolescents’ aggressive behaviors.

In this study, we utilized polynomial regression and response surface analysis (Edwards, 2002). The equation used was: Z = b_0_ + b_1_X + b_2_Y + b_3_X^2^ + b_4_XY+ b_5_Y^2^ + e. Here, Z represents aggressive behavior, X indicates family SES, Y indicates school SES, X^2^ represents the square of family SES, XY represents the interaction between family SES and school SES, and Y^2^ represents the square of school SES. The coefficients b_0_ to b_5_ correspond to the intercept and the regression coefficients of X, Y, X^2^, XY, and Y^2^, respectively.

X and Y were aligned on the line of congruence (LOC). The corresponding coefficients were the slope of the line, denoted as a_1_, and the curvature of the line, denoted as a_2_ (Human et al., 2016). If a_1_ is significant and a_2_ is non-significant, it indicates a linear relationship between the LOC and Z. Specifically, a_1_ > 0 indicates that Z increases as both X and Y increase, while a_1_ < 0 indicates that Z decreases as both X and Y increase. If a_2_ is significant, the LOC forms a curved relationship: a_2_ > 0 indicates a U-shaped curve, and a_2_ < 0 indicates an inverted U-shaped curve.

X and Y were mismatched on the line of incongruence (LOIC). The corresponding coefficients were the slope of the line, denoted as a_3_, and the curvature of the line, denoted as a_4_. If a_3_ is significant and a_4_ is non-significant, it indicates that the LOIC is a straight line: a_3_ > 0 means X is greater than Y and Z is higher, while a_3_ < 0 means X is smaller than Y and Z is higher. If a_4_ is significant, the LOIC forms a curved relationship. Specifically, if a_4_ > 0, the LOIC exhibits a U-shaped curve where Z increases as the difference between X and Y increases. Conversely, if a_4_ < 0, the LOIC shows an inverted U-shaped curve where Z increases as the difference between X and Y decreases. In the current study, Hypothesis 1 would be supported if a_1_ shows a significant negative effect. Hypothesis 2 would be supported if a_4_ shows a significant positive effect.

In order to test the moderating effect, a four-step regression was conducted. Model 1 contained the control variable, X, and Y. Model 2 added X^2^, XY, and Y^2^. Model 3 incorporated the moderating variable W. Based on Model 3, five moderating terms (WX, WY, WX^2^, WXY, and WY^2^) were added in Model 4. In this study, we utilized SPSS 25.0, Excel for response surface analysis, and Origin 2021 to analyze the data.

## 3 Result

### 3.1 Descriptive statistics and correlation analysis

Table 1 presents the descriptive statistics for all variables. Family SES was found to be positively correlated with both the parent–child relationship and school SES (*p* < 0.001). However, there was no significant correlation between family SES and aggressive behavior (*p* = 0.77). School SES showed a positive correlation with the parent–child relationship (*p* < 0.001) and a negative correlation with aggressive behavior (*p* < 0.05). The parent–child relationship was negatively correlated with aggressive behavior (*p* < 0.001).

**Table 1:** Descriptive statistics and correlations (*n* = 2,823).

	1	2	3	4	5	6
1. Gender	-					
2. Age	0.03	-				
3. Family SES	−0.04	−0.33^***^	-			
4. School SES	−0.01	−0.46^***^	0.66^***^	-		
5. Parent–child relationship	0.02	−0.21^***^	0.15^***^	0.17^***^	-	
6. Aggressive behavior	−0.10^***^	−0.02	−0.01	−0.08^*^	−0.43^***^	-
*M*	1.54	13.38	−0.36	−0.13	88.28	13.82
*SD*	0.50	1.56	2.25	0.61	14.75	4.52

^*^p < 0.05; ^**^p < 0.01; ^***^p < 0.001.

### 3.2 The result of polynomial regression

After controlling for the age of the students, stepwise regressions were implemented. The results (see Table 2) demonstrated a significant enhancement in the model’s ability to explain aggressive behavior when squared and interaction terms were included (∆*R^2^* = 0.02, *p* < 0.001). The slope along the LOC was negative and statistically significant (a_1_ = −0.14, *p* < 0.001), indicating that adolescents’ aggressive behavior decreases as both family SES and school SES increase, thereby supporting Hypothesis 1. The curvature of the LOC was positive and statistically significant (a_2_ = 0.10, *p* < 0.05), suggesting a U-shaped relationship. Meanwhile, the slope along the LOIC was positive and statistically significant (a_3_ = 0.23, *p* < 0.001), indicating that family SES exceeds school SES, resulting in higher levels of aggressive behavior among adolescents. The curvature of the LOIC was non-significant (a_4_ = 0.02, *p* = 0.64), indicating a linear association with adolescents’ aggressive behavior along the LOIC.

**Table 2:** The results of polynomial regression and moderating analyses.

	Aggressive behavior					
	Model 1	Model 2	Model 3	Model 4	Model 5	Model 6
Intercept	0.62	0.42	1.01	0.87	0.70	0.69
Control variable						
Age	−0.07^***^	−0.06^**^	−0.12^***^	−0.11^***^	−0.06^**^	−0.06^**^
Independent variable						
X	0.05^*^	0.03	0.05^*^	−0.51^***^	0.03	−0.05
Y	−0.17^***^	−0.16^***^	−0.12^***^	−0.54^***^	−0.16^***^	−0.15^*^
X^2^		−0.05	−0.03	0.06	−0.05	−0.04
XY		0.04	0.01	0.07	0.04	0.17
Y^2^		0.12^***^	0.12^***^	0.69^***^	0.11^***^	0.02
W_1_			−0.46^***^	−0.38^***^		
X*W_1_				−0.46^**^		
Y*W_1_				0.46^***^		
X^2^*W_1_				−0.60^***^		
XY*W_1_				−0.06		
Y^2^*W_1_				−0.07		
W_2_					−0.10^***^	−0.10^**^
X*W_2_						0.09
Y*W_2_						−0.01
X^2^*W_2_						−0.01
XY*W_2_						−0.14
Y^2^*W_2_						0.10
*R^2^*	0.02	0.04	0.23	0.24	0.05	0.05
Δ*R^2^*	0.02^***^	0.02^***^	0.19^***^	0.02^***^	0.01^***^	0.001
LOC (X = Y)						
a_1_		−0.14^***^				
a_2_		0.10^*^				
LOIC (X = –Y)						
a_3_		0.23^***^				
a_4_		0.02				

X = Family SES, Y = School SES, X^2^ = the square of family SES, XY = the interaction between family SES and school SES, Y^2^ = the square of school SES, W_1_ = Parent–child relationship, W_2_ = Gender.

**Figure 1:**
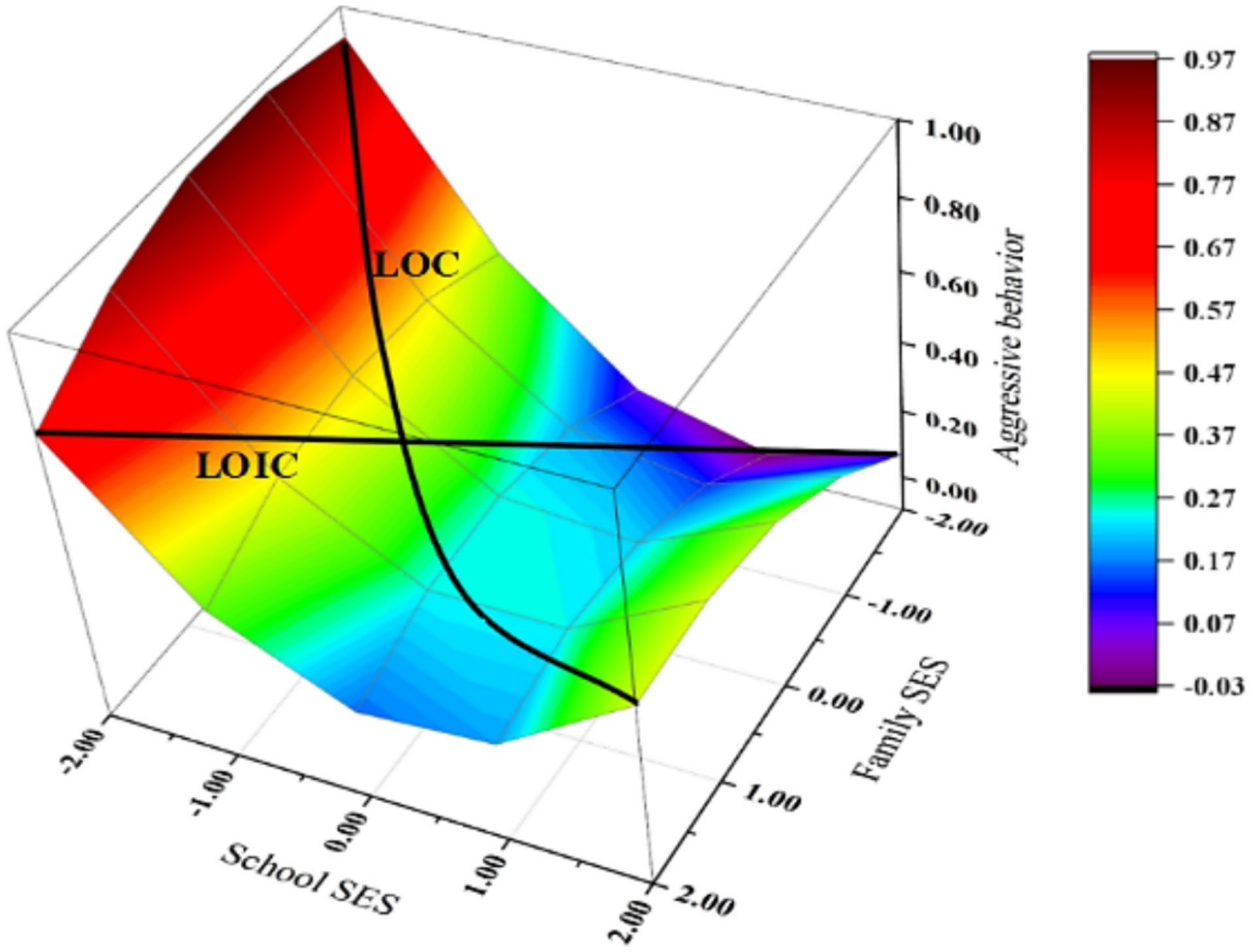
Response surface analysis of the family and school SES predicting adolescents’ aggressive behavior.

Figure 1 was plotted as the response surface based on the data in Table 2. The line extending from the right front corner to the left back corner of the cube is termed the LOC, while the line extending from the right back corner to the left front corner is known as the LOIC. Integrating the data from Table 2 with Figure 1 reveals that the LOC projected onto the response surface forms a U-shaped curve with elevated sides and a depressed center. This study indicates that adolescents with the “LF-LS” SES exhibit more aggressive behaviors compared to those with the “HF-HS” SES. Aggressive behaviors were minimized when both family SES and school SES were at moderate levels. The LOIC projected onto the response surface approximated a straight line, indicating that adolescents with “HF-LS” SES exhibit more aggressive behaviors compared to those with “LF-HS” SES.

### 3.3 The moderating effect of parent–child relationship and gender

**Figure 2:**
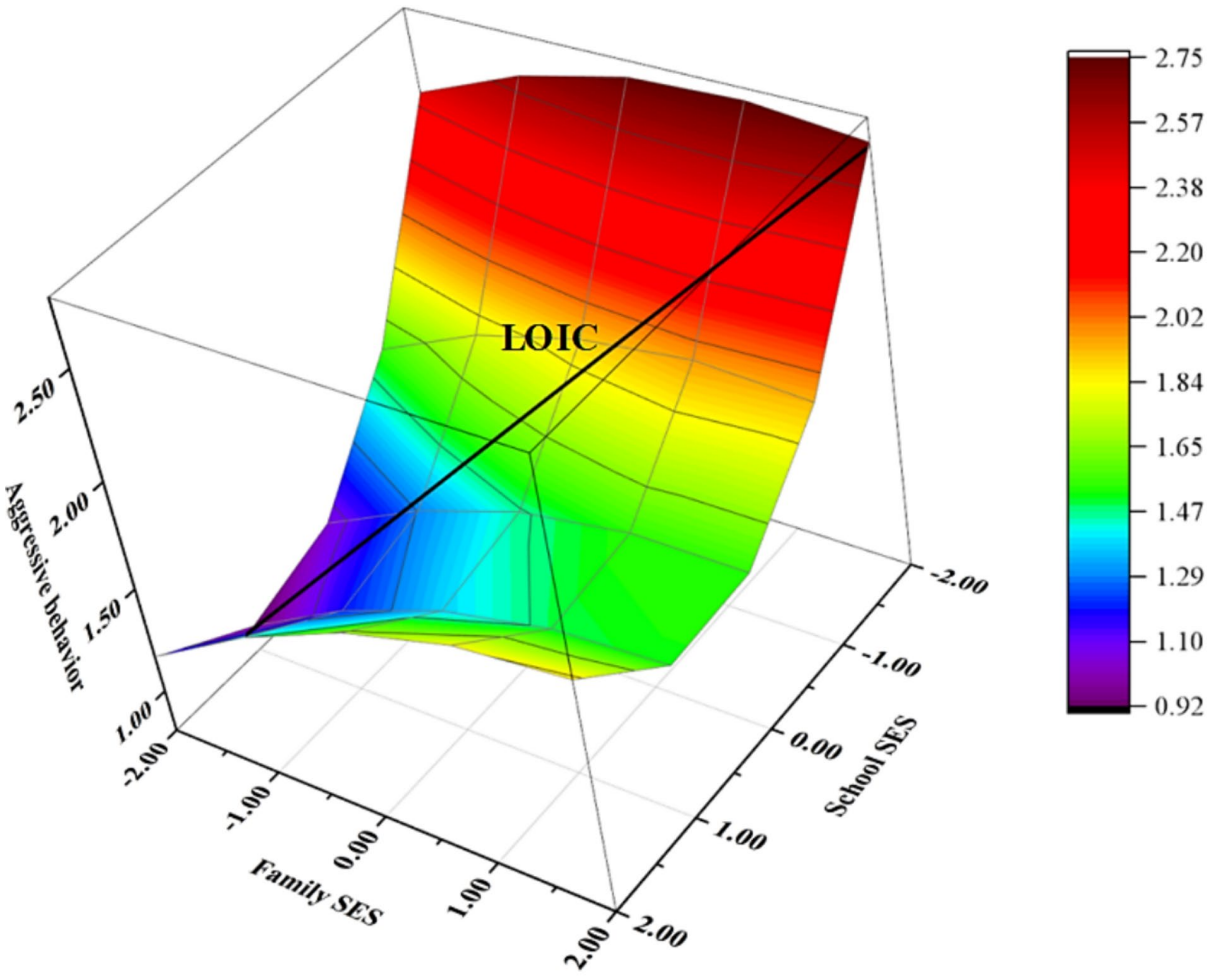
Response surface analysis of low parent–child relationship.

Model 1 to 4 in Table 2 represented the complete steps of analyzing the moderating effect of the parent–child relationship. Model 4 showed a significant increase in ∆*R^2^* (∆*R^2^* = 0.02, *p* < 0.001) with the inclusion of the moderating variable (parent–child relationship), indicating its moderating role. Following the suggestion of Aiken and West (1991), separate polynomial regressions were conducted to test hypothesis 4. The means of the parent–child relationship, with one standard deviation added and subtracted, were analyzed (Table 3), and response surface plots were generated (Figure 2).

**Table 3:** Polynomial regression of high and low parent–child relationship.

	High parent–child relationship		Low parent–child relationship	
	Model 1	Model 2	Model 1	Model 2
Intercept	−0.26	−0.23	2.77	2.30
Control variable				
Age	−0.07	−0.08	−0.21^***^	−0.18^***^
Independent variable				
Family SES	0.04	0.05	0.10^*^	0.08
School SES	−0.04	−0.09	−0.28^***^	−0.22^***^
Family SES^2^		−0.07		−0.003
Family SES × School SES		0.06		−0.03
School SES^2^		0.07		0.18^***^
*R^2^*	0.005	0.013	0.10	0.12
Δ*R^2^*	0.005	0.008	0.10^***^	0.02^*^
LOC (Family SES = School SES)				
a_1_				−0.19
a_2_				0.14
LOIC (Family SES = –School SES)				
a_3_				0.44^***^
a_4_				0.23

The dependent variable is aggressive behavior.

As shown in Table 3, adolescents’ aggressive behaviors were not significantly predicted by either family SES or school SES when the parent–child relationship was high. The inclusion of the squared and interaction terms did not notably enhance the model’s explanatory power (∆*R^2^* = 0.008, *p* = 0.33). Furthermore, the polynomial model was not supported. Conversely, when the parent–child relationship was low, the LOIC score for a_3_ = 0.44, with a significance level of *p* < 0.001. Combined with Figure 2, it is clear that adolescents exhibited higher levels of aggressive behaviors when family SES exceeded school SES.

Model 1 to 6 in Table 2 represented the comprehensive steps of the gender moderating effect analysis. Model 6 showed a non-significant ∆*R^2^* (∆*R^2^* = 0.001, *p* = 0.64) upon including the moderating variable (gender), indicating an absence of significant gender moderation.

## 4 Discussion

Family and school SES are two critical factors influencing adolescents’ aggression. In this study, we investigated the effect of family-school SES match and mismatch on adolescents’ aggressive behaviors using polynomial regression and response surface analysis.

### 4.1 The effect of family-school SES match and mismatch on aggressive behavior

Our study found that aggressive behaviors were higher among adolescents with “LF-LS” SES compared to those with “HF-HS” SES. Hypothesis 1 was supported by these findings. Low family and school SES are identified as risk factors influencing adolescents’ aggressive behaviors (Greitemeyer and Sagioglou, 2016; Xie and Zhang, 2018). The family investment model (Bradley and Corwyn, 2002) suggests that families with low SES lack adequate resources to support their children, potentially contributing to increased aggressive behaviors among these children. Additionally, according to the “context-process-outcomes” model, schools with high SES typically provide superior facilities and foster a harmonious climate (Scheerens, 2011; Xue et al., 2020), which are strongly associated with students’ behavior. Conversely, schools with low SES often lack advanced educational opportunities, and students in these schools are more likely to be influenced negatively by their peers. Consequently, students with “LF-LS” SES exhibit more aggressive behaviors.

However, it was found in our study that aggressive behavior was lowest when adolescents had moderate levels of family and school SES. A study by Aslund et al. (2013) also found a U-shaped relationship between family SES and delinquency. According to the challenge model of psychological resilience, adolescents exposed to moderate levels of risk factors are more likely to adapt to their environment. In conclusion, the least aggressive behaviors among adolescents are observed when their family and school SES are moderate. Nonetheless, further validation of this inference is warranted. Future studies could replicate this experiment to confirm these findings.

When there was a family-school SES mismatch, higher levels of aggressive behaviors were observed among students with “HF-LS” SES compared to those with “LF-HS” SES. The findings only partially supported Hypothesis 2. According to social hierarchy theory, individuals in higher hierarchical positions may exhibit more aggressive behaviors to maintain their superior status (Huhman, 2006). Some studies also indicate elevated levels of aggression among adolescents from higher family SES backgrounds (Aslund et al., 2013; Li, 2016). Moreover, there are other possible explanations for group identity effects. In diverse peer environments, the identities of group members can influence individual development, affecting students’ school performance (Eccles and Roeser, 2011). The proportions of various groups of students in schools influence the power that each group has. There is an imbalance of power between minority groups and mainstream groups in schools (Juvonen et al., 2006), leading to certain levels of segregation, negative interactions, or discrimination. Children from high socioeconomic status families may develop a sense of superiority when surrounded by peers from less advantaged backgrounds, which could contribute to aggressive behavior. Another possible reason could be that only objective socioeconomic status was measured in this study. Research has shown that subjective social status (SSS) may have a stronger predictive effect on aggressive behavior than SES alone (Greitemeyer and Sagioglou, 2016). Therefore, future studies should explore the combined influence of SES and SSS on adolescents’ development to provide further insights.

### 4.2 The moderating role of gender and parent–child relationship

Contrary to Hypothesis 3, there were no significant gender differences in the effect of family-school SES match on aggressive behaviors. This suggests that both boys and girls are equally sensitive to family SES and school SES. The findings underscore the importance of ensuring equal attention to the physical and mental health of both genders in the educational process. Although our questionnaire measured both direct and indirect aggression, we only utilized the data on total aggression for our analysis. Given that boys tend to exhibit more direct aggression and girls more indirect aggression (Ostrov and Godleski, 2010; Chen et al., 2019), these distinctions may not be apparent when considering total aggression alone. In this study, focusing solely on total aggressive behavior may obscure potential gender moderation effects. Furthermore, previous research exploring the relationship between children’s aggressive behavior and family environment have not identified significant differences between boys and girls in aggression. However, both boys’ and girls’ aggressive behaviors appeared to be associated with the family environment (Liu, 2003). This suggests that the influence of family factors on individual aggressive behavior is widespread and cannot be ignored. Therefore, future research could explore the impact of family-school SES match and mismatch on direct and indirect aggressive behaviors separately to yield deeper and different findings.

Consistent with Hypothesis 4, it was found in our study that the parent–child relationship played a moderating role. The effects of family and school SES on aggressive behavior were not significant when the parent–child relationship was strong. This suggests that the parent–child relationship is an important protective factor for adolescents’ aggressive behaviors, aligning with prior research findings (Tian et al., 2018; Yu et al., 2018). As a vital component of the social support system, a family environment characterized by a strong parent–child relationship serves as a comforting arena where adolescents feel accepted and secure (Simmons et al., 1987).

### 4.3 Implications and limitations

This study has several important implications. Firstly, it represents the exploration of the correlation between family-school SES match and adolescents’ aggressive behaviors, thereby enriching existing research on aggressive behavior theoretically. Secondly, this study contributes to earlier research on family-school SES match by offering a better understanding of the relationship between matching or discrepancy in family and school socioeconomic status and aggressive behavior. Thirdly, we utilized polynomial regression and response surface analysis to identify a significant relationship between the socioeconomic status match between families and schools and adolescents’ aggressive behaviors. Fourthly, we investigated whether gender and the parent–child relationship play moderating roles in the effect of the family-school SES match on aggressive behavior, contributing to a deeper understanding of these dynamics. Finally, the research conclusions of this article offer guidance to parents, school administrators, and education officials. Given the match between family and school socioeconomic statuses, parents should avoid pushing their children into schools that do not align with their own socioeconomic backgrounds. School administrators and education policymakers should pay more attention to the relationship between students’ family socioeconomic status and their school socioeconomic status, as well as develop and implement more scientifically grounded educational programs and policies to foster students’ holistic development.

However, the present study has several limitations. Firstly, cross-sectional data were used in this study, which is unable to determine the causal relationship between family-school SES match and aggressive behavior. Future studies could explore the use of longitudinal data for this purpose. Secondly, family SES was assessed using parental education and income in this study. Moreover, school SES was assessed using mean household SES. However, several studies suggest that household resources (e.g., computers and books) can also be indicators of family SES, and school SES can be estimated using school resources (e.g., education funding) (OECD, 2013; Takashiro, 2017). SES could be evaluated using more comprehensive factors in future research. Thirdly, this study utilized a questionnaire method, which may have introduced social approval bias. Future research on aggressive behavior could benefit from using experimental methods.

## 5 Conclusion

The current study shows that adolescents with “Low Family-Low School” SES exhibit more aggressive behaviors compared to those with “High Family-High School” SES. When there is a mismatch between family SES and school SES, students with “High Family-Low School” SES exhibit higher levels of aggressive behavior compared to those with “Low Family-High School” SES. There is no significant difference between boys and girls. Additionally, this study suggests that a strong parent–child relationship can mitigate the impact of family-school SES mismatch on aggressive behavior.

## Data availability statement

The raw data supporting the conclusions of this article will be made available by the authors, without undue reservation.

## Ethics statement

The studies involving humans were approved by the ethics committee of Shanxi university. The studies were conducted in accordance with the local legislation and institutional requirements. Written informed consent for participation in this study was provided by the participants’ legal guardians/next of kin.

## Author contributions

FL: Conceptualization, Funding acquisition, Methodology, Resources, Supervision, Writing – original draft, Writing – review & editing. YW: Data curation, Formal analysis, Validation, Visualization, Writing – original draft, Writing – review & editing. XW: Data curation, Formal analysis, Writing – review & editing.
